# Lipid nanoparticle-based mRNA delivery systems for cancer immunotherapy

**DOI:** 10.1186/s40580-023-00385-3

**Published:** 2023-08-07

**Authors:** Jieun Han, Jaesung Lim, Chi-Pin James Wang, Jun-Hyeok Han, Ha Eun Shin, Se-Na Kim, Dooyong Jeong, Sang Hwi Lee, Bok-Hwan Chun, Chun Gwon Park, Wooram Park

**Affiliations:** 1 Department of Integrative Biotechnology, College of Biotechnology and Bioengineering, Sungkyunkwan University, Seobu-ro 2066, Suwon, Gyeonggi 16419 Republic of Korea; 2 Institute of Biotechnology and Bioengineering, College of Biotechnology and Bioengineering, Sungkyunkwan University, Seobu-ro 2066, Suwon, Gyeonggi 16419 Republic of Korea; 3 Department of Biomedical Engineering, SKKU Institute for Convergence, Sungkyunkwan University (SKKU), Seobu-ro 2066, Suwon, Gyeonggi 16419 Republic of Korea; 4 Department of Intelligent Precision Healthcare Convergence, SKKU Institute for Convergence, Sungkyunkwan University, Seobu-ro 2066, Suwon, Gyeonggi 16419 Republic of Korea; 5 MediArk, Chungdae-ro 1, Seowon-gu, Cheongju, Chungcheongbuk 28644 Republic of Korea; 6 R&D center of HLB Pharmaceutical Co., Ltd., Hwaseong, Gyeonggi 18469 Republic of Korea; 7 Biomedical Institute for Convergence at SKKU (BICS), Sungkyunkwan University, Seobu-ro 2066, Suwon, Gyeonggi 16419 Republic of Korea

**Keywords:** Lipid nanoparticles (LNPs), Messenger RNA (mRNA), Cancer immunotherapy, Tumor-associated antigens (TAAs)

## Abstract

Cancer immunotherapy, which harnesses the power of the immune system, has shown immense promise in the fight against malignancies. Messenger RNA (mRNA) stands as a versatile instrument in this context, with its capacity to encode tumor-associated antigens (TAAs), immune cell receptors, cytokines, and antibodies. Nevertheless, the inherent structural instability of mRNA requires the development of effective delivery systems. Lipid nanoparticles (LNPs) have emerged as significant candidates for mRNA delivery in cancer immunotherapy, providing both protection to the mRNA and enhanced intracellular delivery efficiency. In this review, we offer a comprehensive summary of the recent advancements in LNP-based mRNA delivery systems, with a focus on strategies for optimizing the design and delivery of mRNA-encoded therapeutics in cancer treatment. Furthermore, we delve into the challenges encountered in this field and contemplate future perspectives, aiming to improve the safety and efficacy of LNP-based mRNA cancer immunotherapies.

**Graphical Abstract:**

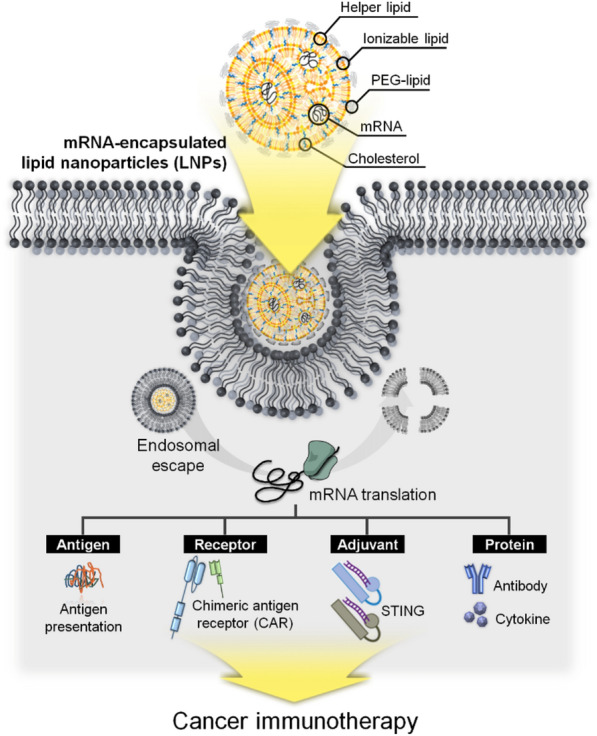

## Introduction

Cancer remains a major health burden worldwide, and the development of effective and safe cancer therapies is an ongoing priority [1]. Cancer immunotherapy has emerged as a promising approach to treat various malignancies by stimulating the patient’s immune system to recognize and eliminate cancer cells [2–4].

**Fig. 1:**
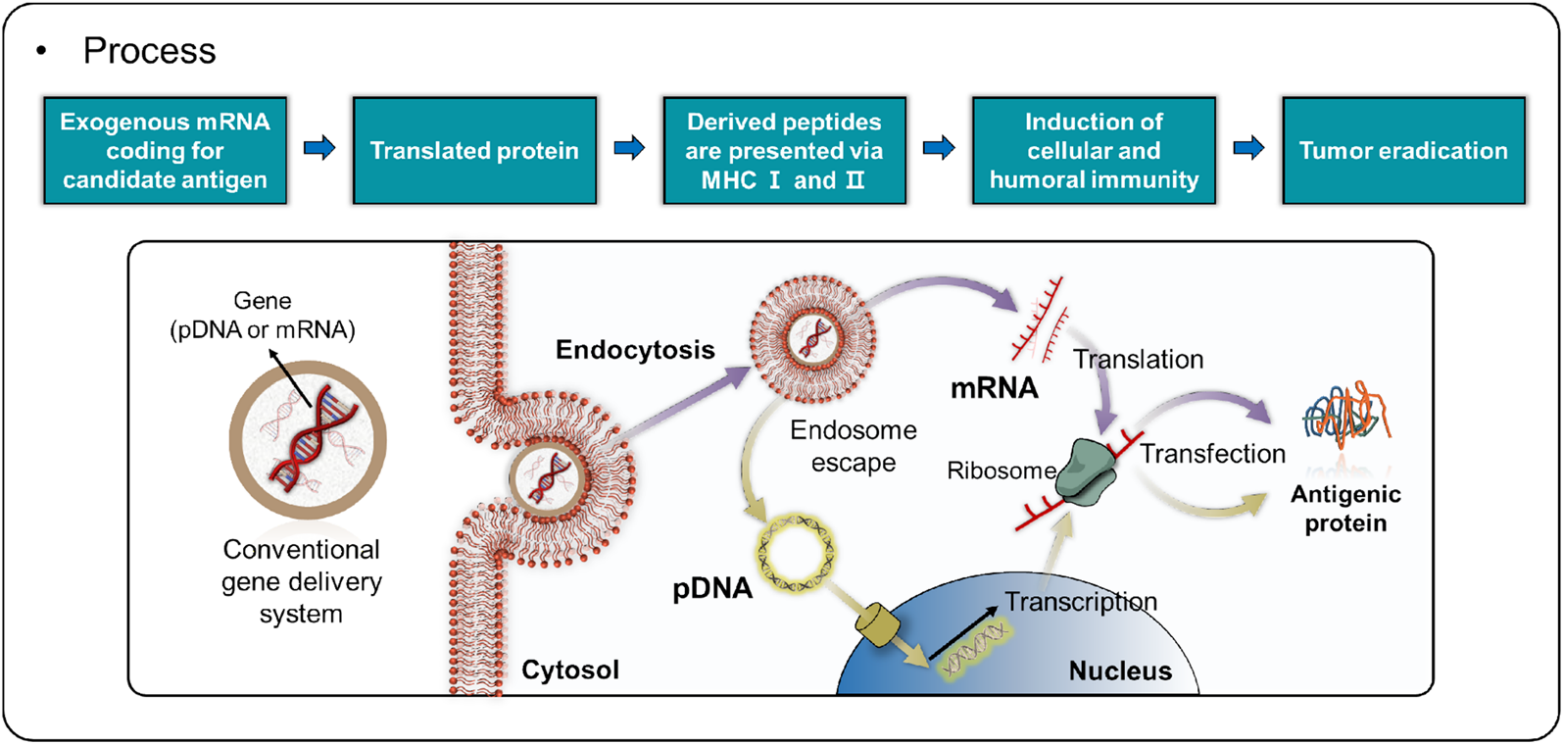
Schematic of the gene delivery process, highlighting pDNA and mRNA pathways. The diagram illustrates the utilization of exogenous mRNA encoding a target antigen, which results in protein translation and peptide presentation through MHC Class I and II molecules. This process activates both cellular and humoral immune responses, ultimately contributing to tumor elimination

Among the various strategies employed in cancer immunotherapy, the use of messenger RNA (mRNA) to encode tumor-associated antigens (TAAs) [5], immune cell receptors [6], cytokines [7], and antibodies [8] has gained significant attention. mRNA holds great potential as a therapeutic agent, with applications ranging from viral vaccines and protein replacement therapies to cancer immunotherapies and genome editing [9–12]. While the idea of utilizing in vitro transcribed (IVT) mRNA as a therapeutic agent traces back to the 1980s, progress was hindered by challenges such as low stability and immunogenicity in vivo [13, 14]. However, the advent of nucleoside-modified mRNA technology has notably diminished mRNA’s immunogenicity while enhancing its translation efficiency, propelling the advancement of mRNA therapeutics [15, 16]. mRNA has emerged as an attractive therapeutic agent endowed with unique advantages. It functions within the cytoplasm, thereby eliminating the risk of unintentional gene alterations or mutations as observed with plasmid DNA (pDNA) (Fig. 1). Moreover, mRNA exhibits high efficacy in dividing cells and can be synthesized on a large scale, rendering it a compelling candidate for the development of novel therapeutic agents to tackle a range of diseases. Recent studies underscore the significant strides made in the realm of mRNA-based drugs for cancer vaccines and immunotherapy [17–20].

For mRNA to be an effective therapeutic agent, a safe and efficient delivery system that allows the mRNA to enter target cells in vivo is required [17, 21–23]. Since mRNA has a short half-life, a carrier is needed to protect it from enzymatic degradation by RNase. The carrier should also be targetable for specific therapeutic effects, and the mRNA must be able to escape from the endosome to regulate the desired protein. A variety of delivery systems have been developed for mRNA carriers, including polymers, lipids, nanoparticles (NPs), and protein derivatives [24–27].

Lipid nanoparticles (LNPs) have emerged as a promising delivery platform for mRNA in cancer immunotherapy, offering protection from degradation and improved cellular uptake [28]. The recent success of LNP-based mRNA vaccines for coronavirus disease 2019 (COVID-19) has further highlighted their potential in the field of cancer treatment [29]. In this review, we will discuss recent advances in LNP-based mRNA delivery systems for various applications in cancer immunotherapy, including TAA-encoding mRNA vaccines, chimeric antigen receptor (CAR)-engineered immune cells, adjuvants, cytokines, and antibodies. We will also outline the challenges and future perspectives for the development and optimization of LNP-based mRNA cancer immunotherapies, with a focus on enhancing efficacy and safety, identifying novel TAAs, and overcoming potential issues related to immune evasion and resistance.

By providing a comprehensive overview of current research and development in LNP-based mRNA delivery systems for cancer immunotherapy, this review aims to contribute to the understanding of this promising therapeutic approach and facilitate its translation into clinical applications.

## LNPs for mRNA delivery

The first generation of LNPs, known as liposomes, emerged in the 1960s [27]. These liposomes were created in an aqueous environment and featured closed lipid bilayer structures [30]. Owing to their ability to enhance the aqueous solubility of drugs, liposomes were quickly recognized as a promising drug delivery system [27]. As nanotechnology advanced, nanosized liposomes were further developed by functionalizing them with targeting ligands or polymers [31]. This rapid progress in various pharmaceutical fields led to the emergence of the “next generation” liposomes, known as LNPs, which have since played a significant role in mRNA delivery [32].

**Fig. 2:**
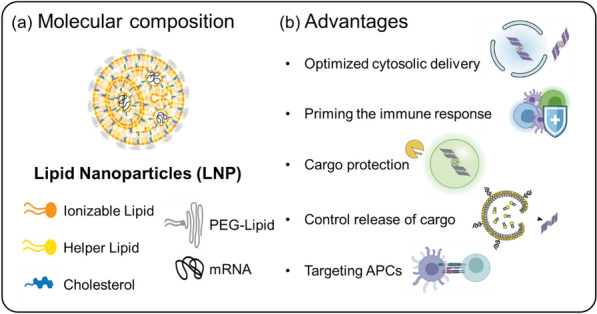
LNPs composition and advantages as gene carriers. **a** Depiction of the molecular composition of LNPs, showcasing a variety of components such as ionizable lipids, helper lipids, cholesterol, polyethylene glycol (PEG)-lipids, and the cargo gene (mRNA). **b** Advantages: LNPs provide several benefits as gene carriers, including enhanced cytosolic delivery, immune response priming, cargo protection, controlled cargo release, and targeting of antigen-presenting cells (APCs)

Recent developed LNPs for mRNA delivery are composed of ionizable lipids, helper lipids, cholesterol, polyethylene glycol (PEG)-lipids, and mRNA (Fig. 2a). LNPs have been investigated as drug delivery systems for encapsulating small molecules, nucleic acids, small interfering RNA (siRNA), and mRNA [14, 33–36]. Owing to advancements in ionizable cationic lipids, LNPs have recently been employed for mRNA delivery [37]. Ionizable lipids are characterized by their pH sensitivity, transitioning from a positively charged state at low pH to neutrality at physiological pH, due to the capacity of their head groups to transfer charge [38, 39]. This property allows them to form ‘mRNA-ionized cationic lipid' complexes that stabilize and safeguard mRNA in a pH-dependent manner [10, 40]. As ionizable cationic lipids maintain neutrality in the bloodstream, the release of mRNA from LNPs can be pH-regulated, helping to mitigate systemic toxicity in vivo [41]. Furthermore, in the low pH environment of endosomes, ionizable lipids can protonate, gaining a positive charge that promotes membrane destabilization and eases the escape of nanoparticles from the endosomal compartment [42]. Another critical component, PEG-lipid, significantly impacts the properties of lipid nanoparticles. It ensures prolonged systemic circulation and enhanced stability by averting opsonization and phagocytosis by macrophages [43, 44]. The choice of PEG-lipid, dependent on PEG molar mass and lipid length, can influence overall outcomes, including targeted delivery and cellular uptake efficiency [45, 46]. Helper lipids and cholesterol are instrumental in LNP formation, governing their fluidity or rigidity [47]. Cholesterol, in particular, also affects the delivery effectiveness and distribution of lipid nanoparticles, with specific modifications amplifying efficacy and selectivity for certain cell types [48, 49].

LNPs have been successfully optimized as vehicles for mRNA delivery and have entered clinical trials for infectious diseases, including Zika virus, chikungunya virus, and influenza [50, 51]. Recently approved COVID-19 vaccines, which are representative of LNP-based mRNA therapeutics encoding the SARS-CoV-2 spike protein, have demonstrated approximately 95% treatment efficacy [34, 36, 52]. As previously mentioned, the ionizable group within LNPs permits protonation at early endosomal pH (approximately pH 6.5), facilitating optimal cytosolic delivery of the cargo. These LNPs serve as protective vessels for the cargo until it reaches the target cell, subsequently priming an immune response. Furthermore, LNPs provide controlled release of cargo into target cells and can be specifically directed towards APCs (Fig. 2b) [50, 53, 54]. Therefore, given these advantageous properties, the potential for LNP-based mRNA delivery in cancer immunotherapy is immense, particularly in treating various solid or aggressive tumors.

## Strategies for mRNA-based cancer immunotherapy with LNPs

**Fig. 3:**
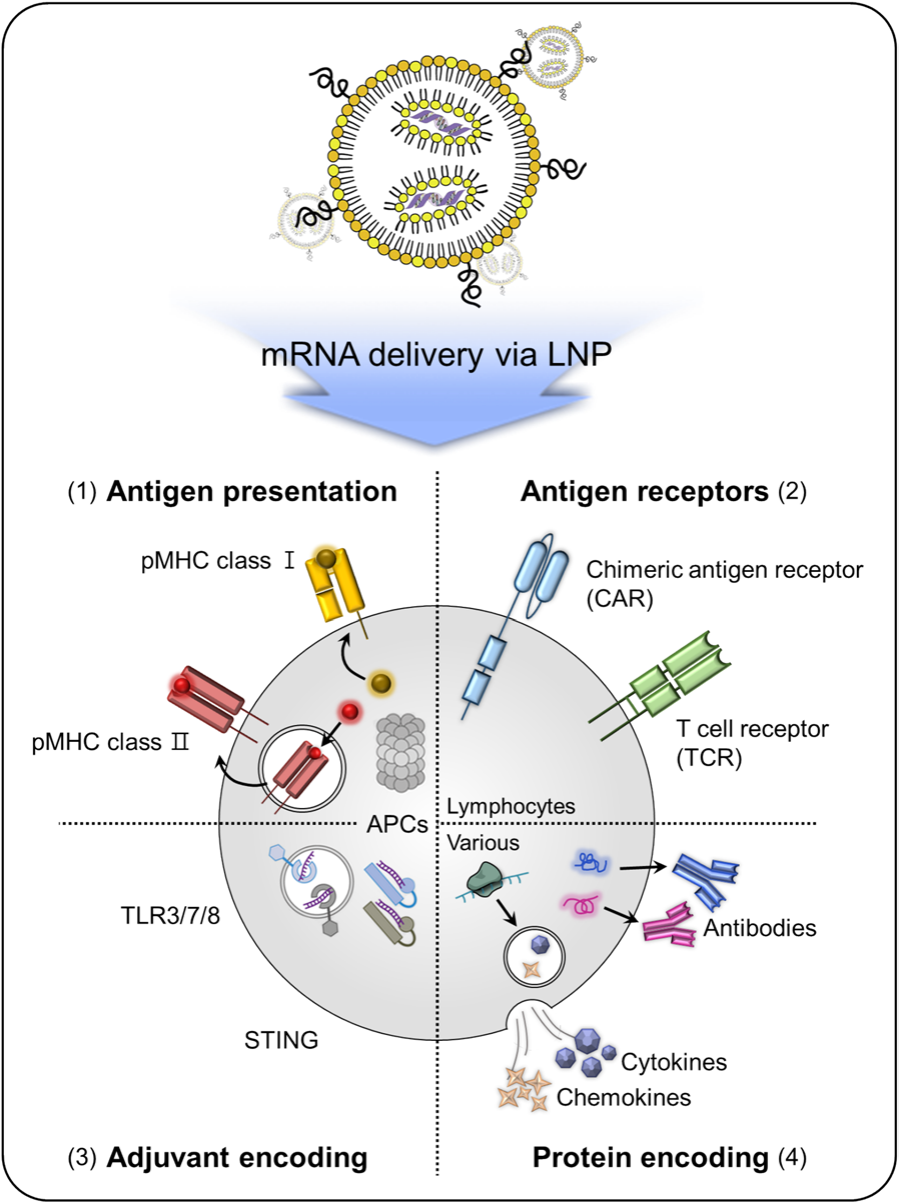
Schematic of strategies for LNP-based mRNA delivery. This diagram displays various approaches to LNP-based mRNA delivery, including: (1) Antigen presentation—delivery of mRNA encoding TAAs to be presented by antigen-presenting cells (APCs); (2) Antigen receptor—delivery of mRNA encoding CARs or TCRs for T cell activation; (3) Adjuvant—the mRNA can encode factors that activate TLR3/7/8 or STING, amplifying immune responses; (4) Protein—delivery of mRNA encoding therapeutic proteins, such as cytokines or antibodies, for direct anti-cancer effects

The development of LNP-based mRNA delivery has addressed the current challenges in cancer immunotherapy associated with protein, peptide, and pDNA delivery. LNP-based mRNA cancer immunotherapy employs four primary approaches: (1) activating immune responses through TAA encoding, (2) expressing antigen receptors such as CAR or T-cell receptors (TCR) encoding, (3) stimulating immunity using adjuvant encoding, and (4) encoding immune-related proteins (*e.g.,* cytokines, antibodies) (Fig. 3). Notably, these LNP-based mRNA strategies have facilitated clinical trials for genetic diseases and cancer treatments (Table 1). Here, we will discuss the progress of LNP-based mRNA delivery trends in cancer immunotherapy and evaluate their treatment efficacy.

**Table 1:** Representative clinical trials of LNP-based mRNA for cancer vaccine

Type of cancer	Name	Encoding	Strategy	References (NCT Number)
Melanoma	mRNA-4157	Personalized 20 TAAs	Monotherapy	NCT03897881
Solid Tumor			Monotherapy or combination with Pembrolizumab (αPD-1)	NCT03313778
Melanoma	Lipo-MERIT	TAAs (NY-ESO-1, Tyrosinase, MAGE-A3, and TPTE)	DC targeted and type I IFN-dependent immunotherapy	NCT02410733
Ovarian Cancer	W_ova1	3 TAAs	Combination with neo-adjuvant chemotherapy (OLIVIA)	NCT04163094
Triple-negative breast cancer (TNBC)	TNBC-MERIT	TAAs	Patient-specific liposome with RNA tailored to the personalized TAAs (IVAC_W_bre1_uID)	NCT02316457
			De novo synthesized RNAs targeting up to 20 individual tumor mutations (IVAC_M_uID)	
Melanoma, Colon Cancer, Gastrointestinal Cancer, Genitourinary Cancer, Hepatocellular Cancer	NCI-4650	20 different TAAs	Immunization	NCT03480152
Melanoma, Non-Small Cell Lung Cancer (NSCLC), Bladder Cancer, Colorectal Cancer, TNBC, Renal Cancer, Head and Neck Cancer, Other Solid Cancers	Autogene Cevumeran (RO7198457)	Neoantigen	Monotherapy or combination with Atezolizumab (αPD-L1)	NCT03289962
Relapsed/Refractory Solid Tumor Malignancies or Lymphoma, Ovarian Cancer	mRNA-2416	Human OX40L	Monotherapy or combination with Durvalumab (αPD-L1)	NCT03323398
Solid Tumor Malignancies, Lymphoma, TNBC, Head and Neck Squamous Cell Carcinoma, Non-Hodgkin Lymphoma, Urothelial Cancer	mRNA-2752	Human OX40L, IL-23, and IL-36γ	Monotherapy or combination with Durvalumab	NCT03739931
Metastatic Neoplasm	SAR441000	IL-12sc, IL-15sushi, IFNα-2b and GM-CSF	Monotherapy or combination with Cemiplimab (αPD-1)	NCT03871348
Solid Tumor and Cancer	MEDI1191	IL-12	Combination with Durvalumab (αPD-L1)	NCT03946800
Neoplasms Carcinoma, NSCLC, Pancreatic Neoplasms, Colorectal Neoplasms	mRNA-5671/V941	G12D, G12V, G13D, and G12C	Monotherapy or combination with Pembrolizumab (αPD-1)	NCT03948763

### Cancer antigen presentation

TAAs are proteins expressed in cancer cells and recognized by the immune system as foreign. Importantly, TAAs are presented to T and B cells by antigen-presenting cells (APCs), which can induce a robust anti-cancer immune response [55, 56]. Several types of cancer immunotherapy that utilize TAAs include cancer vaccines, adoptive cell therapy, and checkpoint inhibitors [57–59]. TAAs have primarily been transferred in the form of whole proteins, antigen peptides, or pDNA encoding specific cancer antigens [5, 60]. Recently, personalized neoantigens have been investigated as dendritic cell (DC) vaccines with potent antitumor effects [61, 62]. In clinical trials, the feasibility of autologous tumor mRNA to elicit an immune response in malignant melanoma has already been evaluated [63]. DCs transfected with TAA-encoding mRNA have emerged as an effective cancer treatment strategy, demonstrating long-term survival rates in clinical trials for brain cancer, prostate cancer, renal cell cancer, and melanoma [64–68]. Notably, 50% of patients with metastatic melanoma who were administered with DC vaccines alone or in combination with interleukin (IL)-2 showed long-term survival without serious adverse effects [68].

**Fig. 4:**
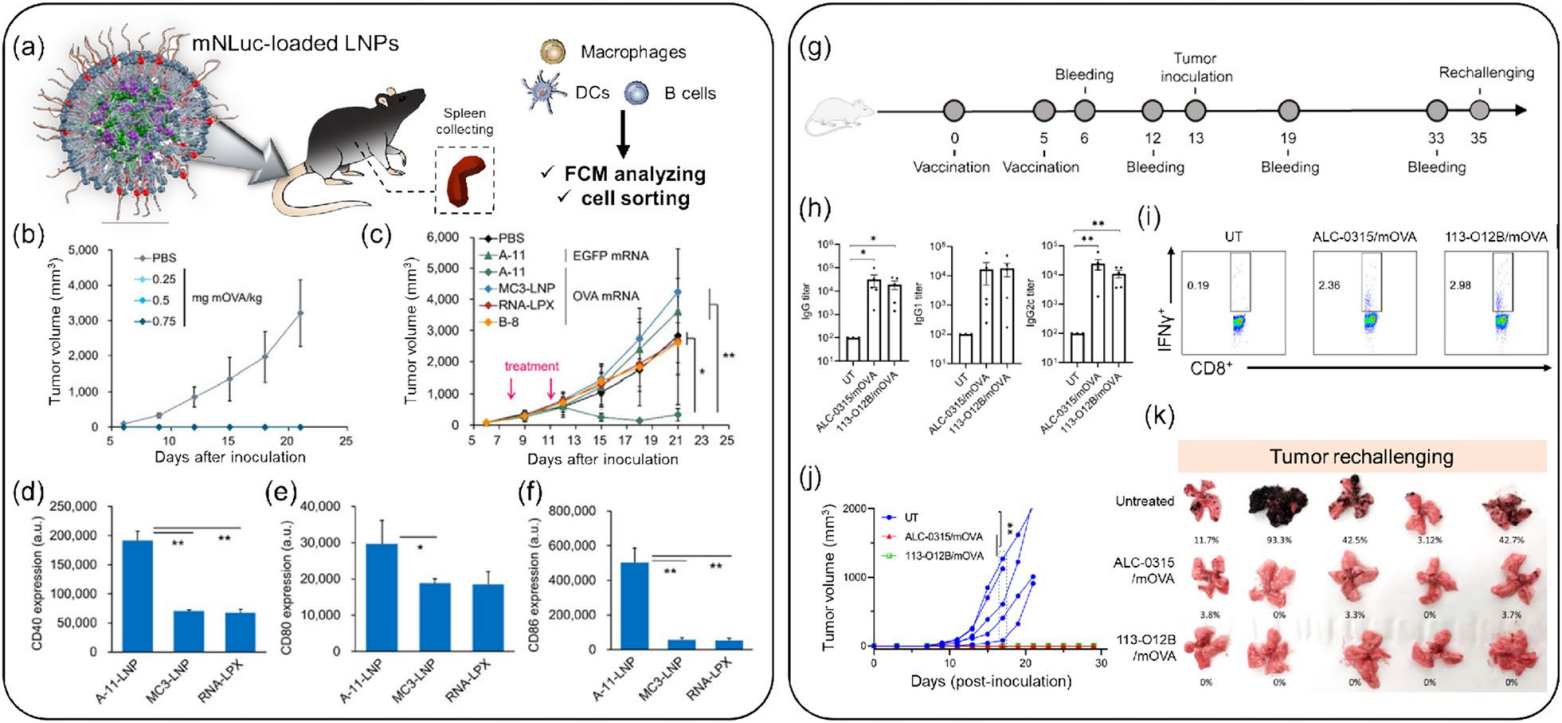
Representative mRNA-LNPs for cancer vaccines. **a** Schematic illustration of the mRNA-loaded LNPs and the experimental method employed. **b** Prophylactic antitumor activity of A11-LNPs in E.G7-OVA tumor-bearing mice. **c** Therapeutic antitumor activity of A-11-LNPs, MC3-LNP, RNA-LPX, and B-8-LNPs in E.G7-OVA tumor-bearing mice, intravenously (i.v.) injected with OVA mRNA-loaded formulations at two doses of 0.03 mg mRNA/kg on days 8 and 11 (n = 5). **d**–**f** Expression of activation markers CD40 (**d**), CD80 (**e**), and CD86 (**f**) in splenic dendritic cells (DCs) 24 h after an i.v. injection of OVA mRNA-loaded formulations at a dose of 0.03 mg mRNA/kg (n = 3). (* *p* < 0.05, ** *p* < 0.01). **a**–**f**: Reproduced from a previous report [70] with Elsevier.) (**g**) Experimental timeline for vaccination and blood withdrawal. **h** OVA-specific antibody titers in mice treated with 113-O12B/mOVA and ALC-0315/mOVA on day 12. (i) Representative flow cytometry diagrams of IFN-γ-positive cells within CD3 + CD8 + T cells 7 days after the second vaccination. **j** Tumor volumes in the B16F10-OVA tumor model. (k) Lungs collected 18 days after the i.v. injection of B16F10-OVA cells. **g**–**k**: Reproduced from a previous report [72] with permission from PNAS)

Given these antitumor effects, mRNA encoding TAAs has emerged as a promising intermediate material for overcoming the limitations of intact TAA delivery. However, since mRNA stability remains a challenge, LNP delivery systems have been actively investigated to enhance mRNA efficacy. Oberli et al*.* optimized LNPs without self-tolerance and demonstrated intracellular delivery of mRNA-encoded TAAs to APCs, promoting cytotoxic CD8 + T-cell responses in a melanoma in vivo model [69]. This LNP-based mRNA delivery system has proven its treatment efficacy, and combination therapy has also shown notable results. In an OVA cancer-bearing mouse model, treatment with LNP-OVA mRNA and C16-R848 effectively suppressed tumor growth (Fig. 4a–f) [70]. Strategies for TAA-encoding mRNA with LNPs continue to improve transfection efficacy by controlling lipid composition. Sasaki et al*.* reported the optimization of LNPs by selecting an appropriate size and lipid composition using a microfluidic device [71]. In this study, the efficacy of A-11-LNP, found to be the optimal formulation, was clinically evaluated by delivering E.G7-OVA mRNA and comparing it with two other LNP formulations. The A-11-LNP group exhibited superior transgene expression activity and maturation in DCs, eliciting a clear therapeutic anti-tumor effect in the E.G7-OVA tumor model.

In recent research, Chen et al*.* reported a lymph-node-targeting mRNA vaccine based on LNPs named 113-O12B for cancer immunotherapy **(**Fig. 4g–k**)** [72]. The targeted delivery of mRNA to the lymph node elicited a robust CD8 + T cell response to the encoded full-length OVA in a B16F10-OVA bearing in vivo model.

### CAR-engineered immune cell

Engineered immune cell therapy has the potential for cancer treatment by enabling specific recognition of cancer cells [73]. A classic example is activating cell-mediated immunity against malignancies by expressing CAR-recognized surface proteins on T cells or natural killer (NK) cells [74, 75]. CAR-T cell cancer therapy began in 2017, following the FDA approval of CD19 CAR-T cells [76]. CAR-encoded mRNA can be delivered to immune cells either ex vivo [77] or in vivo [78], allowing for the expression of CAR on the cell surface and subsequent targeting of cancer cells. Several preclinical and clinical studies have demonstrated the potential of mRNA-encoded CARs for cancer immunotherapy [79]. mRNA-encoded CARs have been transferred via electroporation (EP) [80], or NPs [81], and research is ongoing to develop a platform that is both safer and more efficient. LNPs have been suggested as a means to address CAR-engineered immune cell delivery [82, 83].

**Fig. 5:**
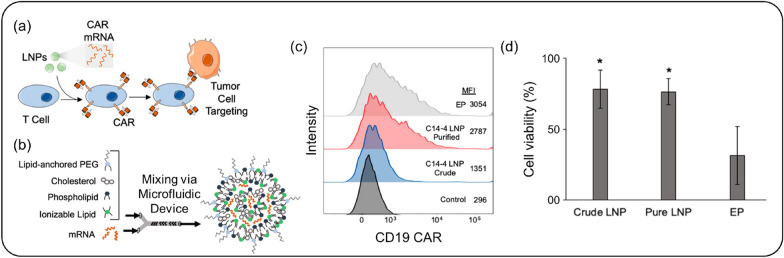
CAR-encoded mRNA-LNP delivery system. **a** Schematic representation of T-cell targeting using CAR mRNA-loaded LNPs. **b** Fabrication of LNPs with various components using microfluidic technology. **c** Expression rate of CAR in primary T cells analyzed by flow cytometry, with both purified LNP and electroporation (EP) groups showing an increase in CAR expression on T cells. **d** Cell viability assessment, highlighting that the EP group exhibited the lowest cell viability among primary T cells. (Reproduced from a previous report [6] with permission from American Chemical Society)

Billingsley et al*.* demonstrated the feasibility of CAR-T cell treatment with an LNP-based mRNA platform by developing ionizable lipids and optimizing an LNP library with various combinations (Fig. 5) [6]. LNPs with the highest transfection efficacy encapsulated the mRNA encoding CD19 CAR and were administered to T cells, expressing CD19 CAR at levels equivalent to or higher than those achieved with electroporation. This novel approach for CAR-T cell immunotherapy shows promise in mRNA delivery and cell engineering technology using LNPs [6, 84]. Given the success of LNP-mediated CAR engineering in T cells, CAR-expressing NK cells have also been investigated for cancer immunotherapy [85, 86]. While efforts have been made to engineer NK cells using LNPs loaded with specific mRNA, most CAR-expressing NK cells have been established using viral vectors rather than LNP-mediated CAR gene delivery [87, 88].

In summary, mRNA-encoded CARs show promise for effective and personalized cancer therapy. However, one notable limitation of mRNA-based CAR engineering is the relatively short duration of gene expression. This transient nature of mRNA-encoded CARs can limit their therapeutic efficacy [80, 83, 89], as the continuous presence of CAR proteins is necessary for sustained immune cell activation and cancer cell elimination. To overcome this challenge, researchers are exploring strategies to improve mRNA stability and extend the duration of CAR expression. These strategies may include modifying the mRNA sequence or structure, optimizing the LNP formulation for enhanced intracellular delivery and release, or developing novel delivery systems that enable sustained or repeated administration of mRNA-encoded CARs [90–92]. Further research is required to optimize the design and delivery of mRNA-encoded CARs, taking into account these improvements to extend gene expression, and to evaluate their safety and efficacy in larger clinical trials. By addressing these limitations and advancing our understanding of mRNA-based CAR engineering, we can enhance the potential of this promising approach in cancer immunotherapy.

### Adjuvant

Immunogenic adjuvants modulate signals contributing to antigen recognition, upregulation of costimulatory molecules, and cytokine production [93]. Toll-like receptors (TLRs) recognize conserved structures present in a wide range of pathogens and trigger innate immune responses, particularly type-I interferon (IFN) production. Due to their ability to connect innate and adaptive immune responses, TLR agonists are highly promising as adjuvants against cancer [94, 95].

The stimulator of interferon genes (STING) is a protein that plays a crucial role in the innate immune response, which is the first line of defense against pathogens such as viruses and bacteria. STING triggers a signaling cascade that activates the transcription factor interferon regulatory factor (IRF)-3 and CD8 + T cell immunity [96]. Due to its central role in the immune response, STING has emerged as a promising agent for the development of cancer immunotherapies [97, 98]. Currently, several STING agonists are in clinical development for the treatment of cancer, aiming to activate the STING pathway and stimulate the immune system to attack tumors [99, 100].

**Fig. 6:**
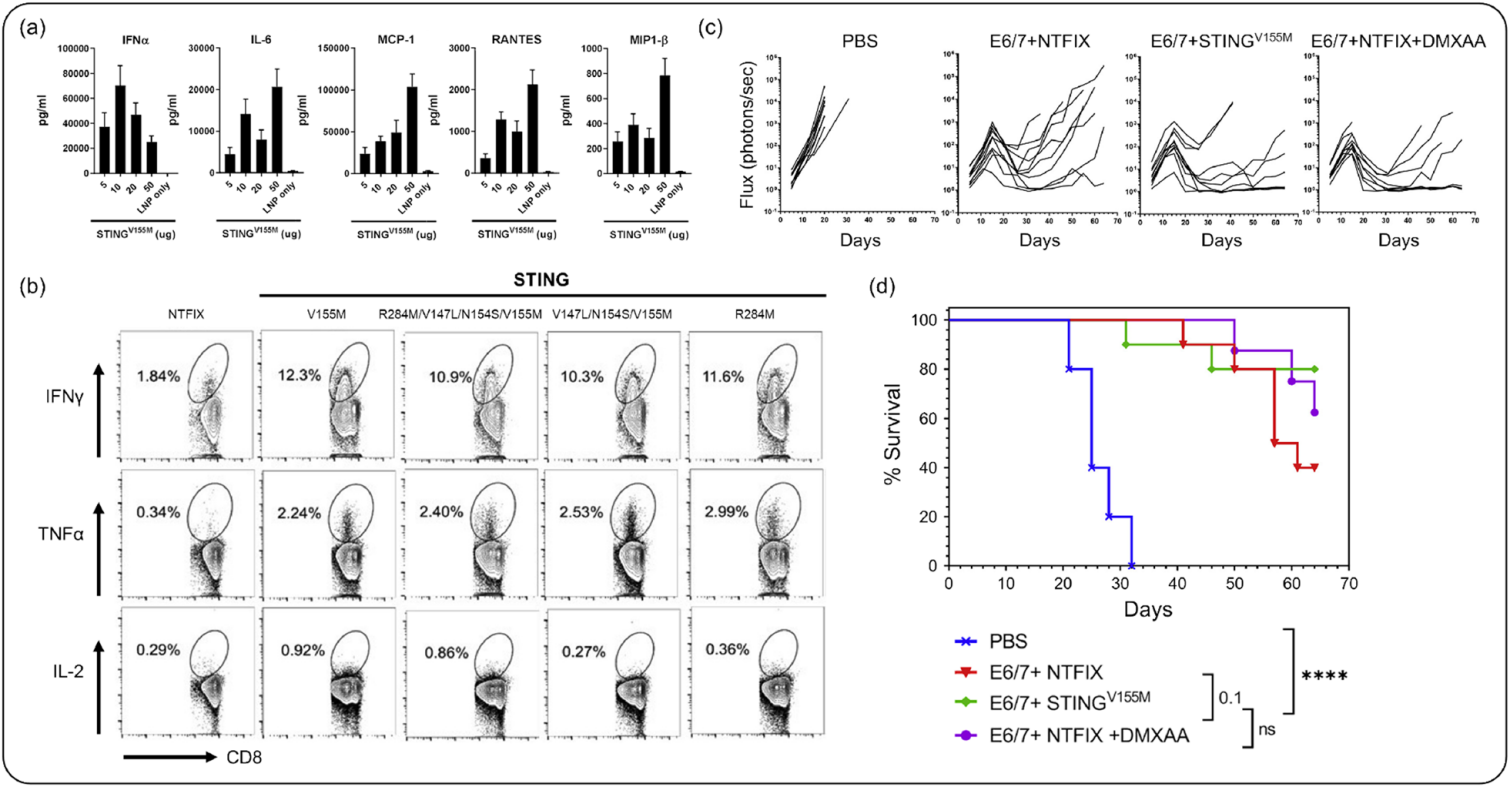
Adjuvant-encoded mRNA-LNP delivery system. **a** Cytokine levels in C57BL/6 mouse serum following treatment with STING-encoded mRNA-LNPs. **b** Flow cytometry analysis of IFN-γ, TNF-α, and IL-2 in the spleen after treatment with STING-encoded mRNA-LNPs. **c** Decreased lung metastasis observed in the E6/7 + NTFIX + DMXAA (STING-encoded mRNA-LNP) group after tumor challenge. **d** Survival rate in mice treated with STING-encoded mRNA-LNPs. (Reproduced from a previous report [101] with permission from Elsevier)

In a study by Tse et al*.*, LNP-encapsulated mRNA vaccines were combined with a genetic adjuvant, a constitutively active mutation of the stimulator of IFN genes (STING^V155M^), to enhance immune responses in preclinical models and clinical studies (Fig. 6) [101]. The adjuvant, which was initially identified in a patient with STING-associated vasculopathy with onset in infancy (SAVI), increased the immunogenicity of vaccines by maximizing CD8 + T cell responses and activating type I IFN pathways through nuclear factor κB (NF-κB) and IFN-stimulated response element (ISRE). When used alongside mRNA vaccines targeting human papillomavirus (HPV) oncoproteins, STING^V155M^ led to reduced tumor growth and increased survival in vaccinated mice, showcasing the potential of mRNA-encoded genetic adjuvants in cancer immunotherapy.

### Cytokine

Cytokines play a key role in regulating the function of the immune system and were among the first cancer treatment medications. IFN-α and IL-2 are representative immunotherapeutic agents for leukemia, metastatic renal cancer, and melanoma [102, 103]. However, due to their short half-lives, large amounts of cytokines had to be administered, which led to systemic toxicity. Although cytokine-encoded pDNA was developed, the expression rate remained low and anticancer efficacy was unclear. To overcome these issues, cytokine-encoded mRNA and delivery systems became a major focus.

**Fig. 7:**
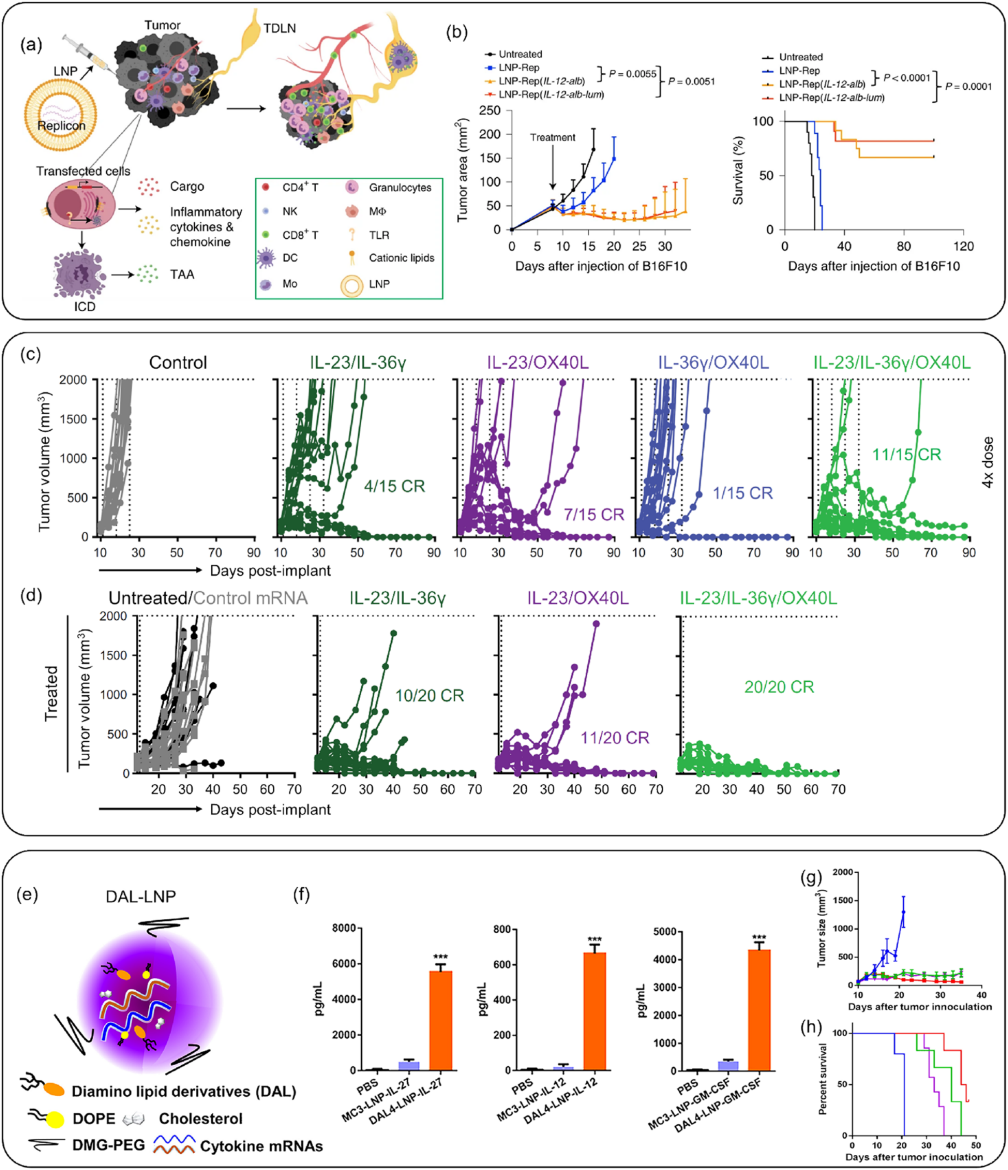
Cytokine-encoded mRNA-LNP Delivery System. **a** Schematic representation of tumor challenge with cytokine-encoded mRNA-LNP systems. **b** Tumor volume and survival rate in the B16F10 tumor model treated with IL-12-encoded mRNA-LNPs. The IL-12-alb-lum mRNA-LNP formulation demonstrated effective tumor suppression efficacy. **a**–**b**: Reproduced from a previous report [106] with permission from Springer Nature). **c** Tumor volume measured for IL-23, IL-36γ, and OX40L-encoded mRNA-LNPs, showing anticancer effects in the MC38-R tumor model. **d** Tumor volume suppression due to the abscopal effect of IL-23, IL-36γ, and OX40L-encoded mRNA-LNPs. **c**–**d**: Reproduced from a previous report [111] with permission from American Association for the Advancement of Science). **e** Schematic of IL-12, IL-27, and GM-CSF-encoded mRNA-LNP systems. **f** Cytokine concentrations following treatment with IL-27, IL-12, or GM-CSF mRNA-loaded MC3-LNPs or DAL4-LNPs. **g** Tumor size measured in the B16F10 murine model treated with DAL4-LNP-encapsulated mRNA. **h** Survival rate in the B16F10 murine model treated with mRNA-loaded DAL4-LNPs. **e**–**h**: Reproduced from a previous report [7] with permission from Elsevier)

IL-12, known as a T cell-stimulating factor, has demonstrated strong anticancer activity in preclinical models but has also caused systemic toxicity after spreading in the blood [104, 105]. Li et al*.* fabricated LNPs encapsulating mRNA simultaneously encoding IL-12 and lumican [106]. Lumican retained the IL-12 within the tumor microenvironment (TME), reducing side effects. The LNPs encapsulated mRNA inducing immunogenic cell death (ICD) were confirmed in vitro and in vivo, resulting in the effective induction of type-I IFN, TLR3, and boosting immunological memory within the TME (Fig. 7a, b).

The IL-1 and IL-12 families cooperate for anti-inflammatory and antitumor immune responses. The IL-1 family (IL-1, IL-18, IL-33, IL-36, IL-37, and IL-38) is involved in the early immune response following antigen invasion [107]. IL-36 is known to be correlated with a good prognosis in cancer and stimulates APCs and T cells [108]. The IL-12 family cytokines serve as a bridge between innate and adaptive immunity [109]. IL-23, a member of the IL-12 family, regulates the immune response and exhibits antitumor effects [110]. Hewitt et al*.* designed OX40, IL-36, and IL-23 encoded mRNA and conducted monotherapy and combination therapy for tumors using an LNP-based delivery system (Fig. 7c, d) [111]. The triplet (OX40, IL-36, and IL-23)-encoded mRNA delivered by LNPs effectively activated DCs and T cells, resulting in significantly enhanced anticancer effects compared to singlet-encoded mRNA. These strategies elicited both innate and adaptive immune responses, preventing tumor recurrence effectively even when the tumor was re-challenged.

In another studies, Liu et al*.* demonstrated anticancer treatments using cytokine-encoded mRNA loaded LNPs **(**Fig. 7e–h**)** [7]. These cytokines (*i.e.,* IL-12, IL-27, and GM-CSF) exhibited synergistic effects, increasing T cell survival in the TME and promoting memory T cells with IFN-γ and IL-10. LNP-based multiple mRNA strategies were evaluated for expression efficacy in a melanoma model, and outstanding tumor suppression was reported without toxicity. The combination of IL-12 and IL-27 attracted B cells, macrophages, CD4 + /CD8 + T cells, and NK cells, demonstrating the potential of multiple cytokine-encoded mRNA with LNP delivery systems to aggregate immune cells and provide effective therapies.

### Antibody

Antibody-based treatments have been widely recognized as effective therapies for conditions such as cancer, chronic inflammation, and autoimmune diseases [112]. In cancer therapy, antibodies not only directly opsonize cancer cells, but also interact with the immune system, triggering both innate and adaptive immune responses [113]. However, despite their clinical success and potential, antibody treatments present certain limitations. Stability issues, the intricacies of large-scale manufacturing, and considerable production and treatment costs can impede their wide and accessible application to all patients [114]. A promising alternative involves the in vivo production of antibodies via delivery of antibody-encoded mRNA, a method that can lead to efficient in vivo expression of desired antibodies [115, 116].

**Fig. 8:**
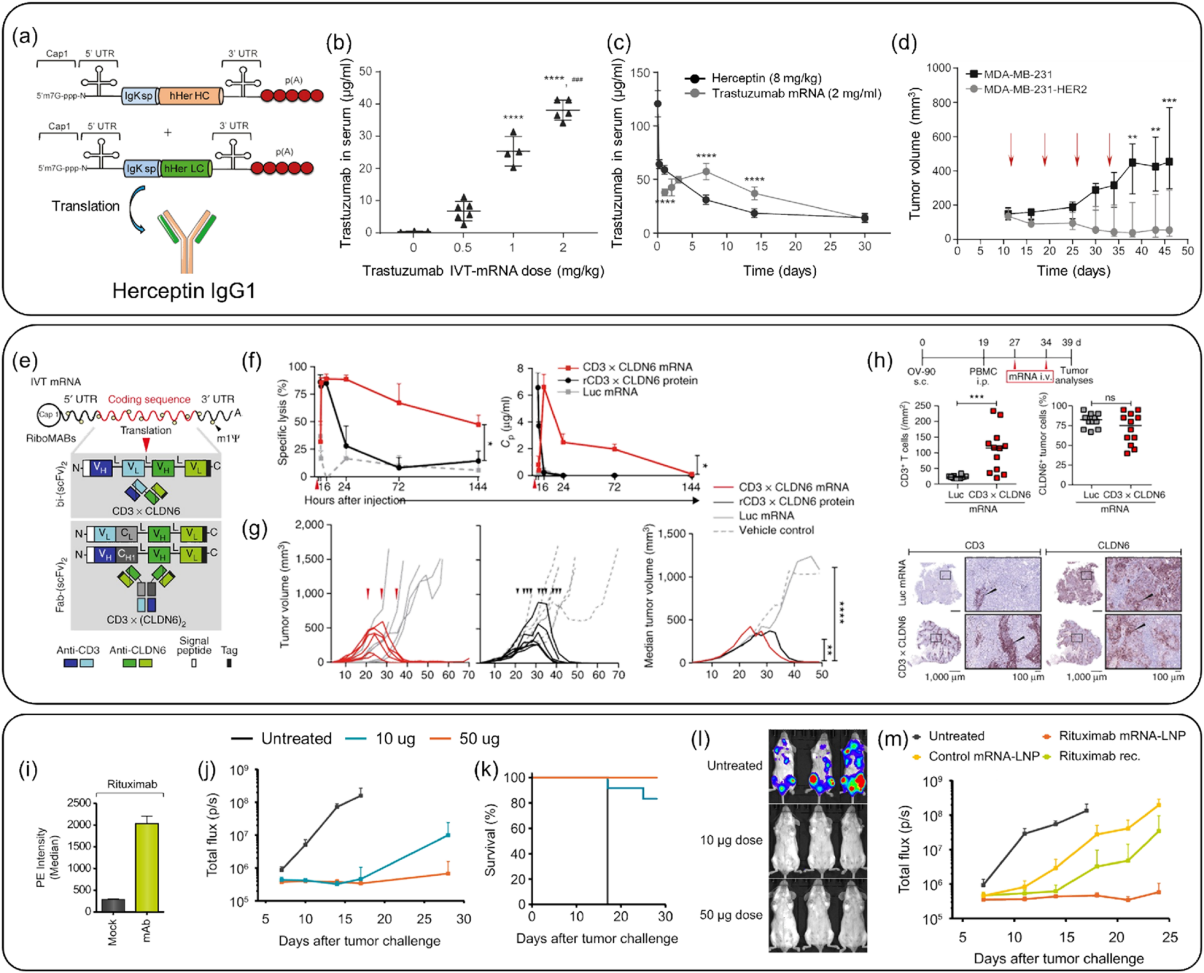
Antibody-encoded mRNA-LNP Delivery System. **a** Schematic representation of mRNAs encoding the heavy and light chains of trastuzumab. **b** Trastuzumab concentrations in C57BL/6 mouse serum 24 h after injection of cKK-E12 LNPs with trastuzumab mRNA via the tail vein at different doses. **c** Pharmacokinetics of trastuzumab in C57BL/6 mouse serum after a single i.v. dose of 8 mg/kg Herceptin (Genentech) or 2 mg/kg cKK-E12 LNPs with trastuzumab mRNA. **d** Growth of HER2-negative (MDA-MB-231) and HER2-positive (MDA-MB-231-HER2) tumors in mice treated with trastuzumab mRNA. Arrows indicate the days of mRNA-LNP injections. **a**–**d**: Reproduced from a previous report [8] with permission from Elsevier). **e** Structures of the IVT bi-(scFv)_2_ and Fab-(scFv)_2_ RiboMABs. **f** Ex vivo cytotoxicity (left) and concentration (Cp) (right) of endogenously translated CD3 × CLDN6 RiboMAB in the plasma of NSG mice after i.v. administration of polymer/lipid-formulated mRNA. **g** Mice were treated with CD3 × CLDN6 or luciferase mRNA (n = 6/group; three doses of 3 µg/mouse i.v. weekly) or with purified CD3 × CLDN6 protein (200 µg/kg) or vehicle (n = 7/group; three doses intraperitoneally (i.p.) weekly, total of ten doses). Tumor growth for individual mice (left, mRNA; right, recombinant protein) are shown. **h** Mice were treated with two doses of CD3 × CLDN6 mRNA (n = 4) or luciferase mRNA as a negative control (n = 4) (both 3 µg/mouse i.v. weekly). Tumor-infiltrating lymphocytes (human CD3 + cells; left) and CLDN6-expressing tumor cells (right) were quantified by immunohistochemistry in three consecutive tumor sections. **e**–**h**: Reproduced from a previous report [121] with permission from Springer Nature). **i** Binding of mRNA-encoded rituximab expressed in BHK cells to Raji cells. Depicted is the median of phycoerythrin (PE) fluorescence of all living cells. **j**–**m** mRNA-encoded mAb protects mice from lethal tumor challenge. Each group comprised 12 mice. **j** Tumor development assessed by whole-body luminescence imaging at indicated times after tumor challenge. **k** Survival of mice receiving i.v. injections of either 10 or 50 µg of mRNA-LNP encoding rituximab. **l** Representative luminescence images of mice treated with two different doses of mRNA-LNP encoding rituximab or untreated mice at day 13 after tumor challenge. **m** Tumor development of mice receiving i.v. injections of 50 µg of mRNA-LNP encoding rituximab or control antibody or 200 µg of recombinant rituximab. The experiment was assessed by whole-body luminescence imaging at indicated times after tumor challenge. (Reproduced from a previous report [122] with permission from EMBO Press)

HER2 antibody (*i.e.,* Trastuzumab) is a well-known example of an antibody-based cancer treatment targeting HER2, which is overexpressed in cancer patients [117, 118]. When trastuzumab binds to HER2 of cancer cells, it shows anticancer effects by blocking the proliferation and survival pathway of cancer cells [119, 120]. Based on this mechanism, Rybakova et al*.* investigated designing and delivering trastuzumab-expressing mRNA with LNP through IVT technology (Fig. 8a–d) [8]. In the group in which trastuzumab-encoded mRNA with LNP was injected into mice, the concentration of trastuzumab expressed in serum gradually increased until after 7 days. On the other hand, the intact form of trastuzumab injected group continued to decrease its level in serum, which means the antibody-encoded mRNA provided the possibility of being an alternative to antibody therapeutics when delivered with LNPs.

Sahin’s group designed mRNAs encoding RiboMABs against the T cell receptor-related molecule CD3 and the tight junction protein Claudin6 (CLDN6), one of TAAs (Fig. 8e–h) [121]. After systemically administering CD3 × CLDN6 RiboMAB into LNPs to mice and measuring its concentration in serum over time, the mRNA gradually decreased over 144 h, while the antibody protein rapidly disappeared 6 h after administration. Administration of CD3 × CLDN6 RiboMAB to an ovarian cancer xenograft mouse model resulted in complete tumor elimination compared to the control and antibody protein treatment groups. These results were supported by the infiltration of T cells activated by CD3 × CLDN6 RiboMAB into the tumor. Taken together, this study showed that systemic administration of low doses of mRNA resulted in sustained antibody production, whereas the corresponding antibody therapeutic agent had a short half-life, resulting in a significant difference in cancer treatment effect. The study highlighted that low dose of LNP-based mRNA, which can be repeatedly administered and reproduced, resulted in sustained antibody production, overcoming the limitations of antibody therapeutics with short half-lives and showcasing its potential clinical applicability.

In another study, Thran et al*.* explored the use of rituximab, a CD20-targeting antibody widely used for lymphoma treatment, and investigated the utility of chemically unmodified mRNA for passive immunization. They designed rituximab-encoded mRNA encapsulated in LNPs and evaluated its antitumor effects (Fig. 8i–m) [122]. In an in vivo lymphoma model, the group treated with rituximab-encoded mRNA in LNPs exhibited higher tumor suppression and survival rates compared to the group treated with the recombinant rituximab antibody. Furthermore, a single injection of mRNA-LNPs was sufficient to achieve rapid, robust, and long-lasting serum antibody titers, providing both prophylactic and therapeutic protection against lethal rabies infection or botulinum intoxication. This mRNA-mediated antibody expression enabled mice to survive otherwise lethal tumor challenges. These findings suggest that antibody-encoded mRNA-LNPs offer better delivery and treatment efficacy than their recombinant protein counterparts and demonstrate the potential of formulated mRNA as a potent novel technology for passive immunization.

## Conclusion and future perspectives

In conclusion, mRNA-based therapeutic strategies have recently garnered considerable attention due to their simplicity in manufacturing and the capability to produce encoded proteins without genomic mutation. However, due to mRNA's inherent structural instability, an efficient vector or delivery carrier is required to enhance endocytosis efficacy. With the advent of nanoparticle-targeted delivery technologies, LNPs have emerged as an innovative delivery platform that improves mRNA stability and intracellular delivery efficacy, positioning them as highly promising candidates for cancer immunotherapy.

LNPs are typically composed of ionizable lipids, cholesterol that modulates lipid bilayer fluidity, PEG-lipids that enhance particle stability, and helper lipids. When formulated with nanoparticles, these constituents protect mRNA from degradation and facilitate its transfer to the cytoplasm of target cells, thus enabling in vivo and in situ expression. Research on LNP-based mRNA delivery has presented advanced results across several therapeutic strategies. As cancer antigens recognized by APCs elicit robust immune responses, the use of TAAs-encoded mRNA has emerged as a promising method, resulting in heightened transfection efficacy and evident therapeutic effects in cancer therapy. Similarly, mRNA encoding CARs, adjuvants, cytokines, and antibodies have also demonstrated the potential to reduce tumor growth, further underscoring the potential of mRNA-based cancer immunotherapy facilitated by LNPs.

LNP-based mRNA delivery has demonstrated robust intracellular delivery efficacy, enhanced endosomal escape, and efficient protein expression at target cells, thereby enabling potent anticancer treatments. Building on these findings, future advancements in LNP-based mRNA delivery systems will likely focus on refining the formulation of LNPs and enhancing their therapeutic efficacy. The COVID-19 pandemic in particular has spurred a wave of nonclinical trials to test and select effective LNP-mRNA formulations, which were then followed up by clinical trials. However, results from these nonclinical trials did not always align with clinical findings, underscoring the need for complementary technologies to bridge this gap and promote successful LNP development.

Further research is necessary to investigate unexplored potential interactions between mRNA and ionizable cationic lipids in LNPs, as well as to understand the impact of impurities that could potentially disrupt the mRNA [123]. Current practice necessitates LNPs storage at – 80 ℃ to preserve their activity, but efforts are underway to develop novel LNPs that can maintain their activity at room temperature. A significant challenge remains in effectively comparing the delivery and distribution of various LNP formulations, particularly in vivo. Barcode nanoparticle technology offers a solution to this issue by enabling the profiling of LNP distribution at the cellular level in vivo [124, 125]. Large-scale data analysis tools capable of deciphering the relationship between LNP properties and biodistribution are poised to accelerate LNP development. In light of recent technological advancements, AI analytics are being increasingly leveraged to enhance the precision and accuracy of these analytical efforts [126, 127].

Various techniques have been established to add functional moieties to the surface of LNPs, thereby enhancing the recognition of specific targets. The utilization of targeted modalities or cell membrane/extracellular vesicle hybrid systems is expected to boost the effectiveness of disease treatments while simultaneously enhancing targetability and intracellular delivery efficiency [128, 129]. Active applications of technologies such as synthetic biology [130, 131], which can engineer cell membranes to target tumors, and click chemistry [132, 133], which can precisely conjugate cancer-targeting ligands to the surface of LNPs, are anticipated.

To surmount the hurdles of immune evasion or resistance, the identification of novel TAAs and the development of innovative strategies will be considered. The inherent modularity of mRNA presents a particularly promising avenue for personalized neoantigen vaccines, as these stimulate an anti-tumor immune response. However, the precise selection of neoantigens still poses a challenge, necessitating the sequencing of the tumor genome, the identification of mutations, and the prediction of mutations likely to result in high-affinity binding of neoantigen peptides to MHCs [134]. The capacity to generate in vitro mRNA that encodes patient-specific neoantigens directly from sequencing data—bypassing the need for ex vivo cell culture or protein engineering—provides significant advantages for neoantigen vaccination. This platform extends several benefits, such as the ability to encode multiple neoantigens within a single mRNA molecule, thereby amplifying the vaccine's potency. While definitive studies on cross-species variations in mRNA delivery efficacy and cellular responses to LNPs are lacking, Hatit et al*.* recently addressed this gap by analyzing these differences and introducing an engineered murine model with predictable clinical outcomes, thereby tackling challenges associated with cross-species discrepancies [70]. Identifying the factors that contribute to low transfection rates in lymphocytes or monocytes, and devising strategies to enhance them, is pivotal for the advancement of LNP-based mRNA delivery systems in cancer immunotherapy. Through the successful implementation of LNP-based mRNA delivery strategies, we can pave the way for the development of next-generation drugs for cancer immunotherapy.

By addressing these challenges and focusing on optimization, LNP-based mRNA cancer immunotherapies hold substantial potential to transform the landscape of cancer treatment. As research and development in this field progress, we anticipate the emergence of more effective, personalized, and safer therapeutic options. Ultimately, these advancements aim not only to enhance treatment outcomes but also to improve the quality of life for those affected by cancer.

## Data Availability

Not applicable.
